# Local Toxicity of Biocides after Direct and Aerosol Exposure on the Human Skin Epidermis and Airway Tissue Models

**DOI:** 10.3390/toxics9020029

**Published:** 2021-02-03

**Authors:** Nahyun Lee, Dae Yong Jang, Do Hyeon Lee, Haengdueng Jeong, Ki Taek Nam, Dal-Woong Choi, Kyung-Min Lim

**Affiliations:** 1 College of Pharmacy, Ewha Womans University, Seoul 03760, Korea; lxxnxhxxx@gmail.com; 2 Department of Public Health Sciences, Transdisciplinary Major in Learning Health Systems, Graduate School, Korea University, Seoul 02481, Korea; dyjang0803@gmail.com (D.Y.J.); key6855@naver.com (D.H.L.); 3 Severance Biomedical Science Institute, Brain Korea 21 PLUS Project for Medical Science, College of Medicine, Yonsei University, Seodaemungu, Seoul 03722, Korea; pwrttony@yuhs.ac (H.J.); kitaek@yuhs.ac (K.T.N.)

**Keywords:** biocides, aerosol, 3D reconstructed model, KeraSkin^TM^, reconstructed human epidermis, SoluAirway^TM^, reconstructed human airway mucosa

## Abstract

Biocides are commonly used as spray- or trigger-type formulations, thus dermal and respiratory exposure to biocide aerosol is unavoidable. However, little is known about the impact of aerosolization on the local toxicity of biocides on the skin or the airway. We compared the local toxicity of biocides after direct or aerosol exposure on reconstructed human skin epidermis and upper airway models. Three biocides, 1,2-benzisothiazol-3(2H)-one (BIT), 2-phenoxyethanol (PE), and 2-phenylphenol (OPP), most widely used in the market were selected. When the biocide was treated in aerosols, toxicity to the skin epidermis and upper airway tissue became significantly attenuated compared with the direct application as determined by the higher tissue viabilities. This was further confirmed in histological examination, wherein the tissue damages were less pronounced. LC-MS/MS and GC/MS analysis revealed that concentrations of biocides decreased during aerosolization. Importantly, the toxicity of biocides treated in 3 μm (median mass aerodynamic diameter (MMAD)) aerosols was stronger than that of 5 μm aerosol, suggesting that the aerosol particle size may affect biocide toxicity. Collectively, we demonstrated that aerosolization could affect the local toxicity of biocides on the skin epidermis and the upper airway.

## 1. Introduction

Biocides are being widely used to disinfect skin, decontaminate surfaces, and preserve products from microbial contamination. However, biocides may cause harmful effects on human health since they are designed to eliminate living organisms. Human can be exposed to biocidal products via direct dermal contact or inhalation during the use of spray- or trigger-type biocides [1]. Especially inhalation exposure to biocides aerosol is considered to be hazardous, as exemplified by a recent catastrophe associated with inadvertent use of a carpet decontaminating biocide, polyhexamethylene guanidine (PHMG), for a humidifier sterilizer [2]. PHMG is highly skin-irritating, but its oral toxicity is low. Unexpectedly, the use of PHMG as a humidifier disinfectant provoked pulmonary fibrosis, claiming hundreds of lives in Korea [3,4]. In addition, due to the outbreak of CoViD-19 pandemic, the role of disinfectants and sanitizers has become critical in the prevention of infection, and the use of them is on the rise worldwide [5], raising an urgent need to assess their inhalation toxicity for the safe use.

In this regard, it is a prerequisite to ensure the safety of biocides when there is any chance of inhalational exposure. However, an animal inhalation test, a gold standard for the safety assessment of aerosols, is difficult to conduct due to prohibitive cost, heavy facility, and resources [6,7]. Furthermore, the animal inhalation test is considered to be unethical. During the test, unreasonably high dose levels of test chemicals are given through the breathing air, which may cause a considerable distress on the animals [8] (to date, scientists have studied and developed the in vitro test method, a sophisticated experiment using human cells and tissues, replacing animals, and are actually using it for diseases and products related to human health).

Indeed, many in vitro test methods have been studied to evaluate the inhalation toxicity of chemicals [9,10,11,12]. A previous study demonstrated that an in vitro three-dimensional human airway model (Epi-Airway^TM^) combined with multiple endpoint analysis (histology, viability, intracellular glutathione (GSH) levels, and mRNA expression) could provide a robust model for evaluating various types of respiratory toxicity. The results correlated very well with known in vivo responses [12]. Another study showed mechanistic similarities between asthmatic models of 3D air–liquid interface (ALI) cultures derived human bronchial epithelia cells and mouse lung tissue. Only 19% of mouse lung genes with human orthologues were not expressed in the 3D ALI model. It demonstrated 3D ALI models based on epithelial cells reduce the gap between traditional 2D in vitro assays and animal models [11]. However, this study directly exposed the tissues to the test chemicals, which could not account for the characteristics of inhalation exposure.

To reflect the aerosol exposure occurring in real-life exposure scenarios, several studies have used an in vitro aerosol exposure system, VITROCELL^®^ (VITROCELL Systems GmbH, Waldkirch, Germany). VITROCELL^®^ is successfully applied to expose aerosolized test chemicals on ALI in vitro. This system enables a uniform deposition of aerosols through monitoring the mass of the deposited aerosol and a constant flow of the conditioned aerosol [10,13]. Additionally, Tollstadius, Bruna Ferreira, et al. evaluated the toxicity of carbendazim, used in agriculture against fungal plant diseases, on A549 alveolar cells both in monolayer and an air–liquid interface cell system with VITROCELL^®^ Cloud 12 chamber. They demonstrated that carbendazim induced cell death in a 3D reconstructed alveolar model, but the toxicity was not observed with the monolayer exposure model [14]. Although the performance of VITROCELL^®^ systems is considered good for the exposures of aerosols, these systems are expensive, and professional expertise is needed for maintenance and cleaning [15,16,17]. Another study investigated the toxicity of aerosolized impregnation products (IP) in vitro using a simple jet nebulizer and a syringe pump. They examined the effects of aerosolized IP on a non-viable lung surfactant droplet system in a constrained drop surfactometry. The sensitivity of this method in predicting acute inhalation toxicity was good (100% (13/13)), but specificity was low (62.5% (5/8)), reflecting the limitation of the non-viable test system [18].

Here, to examine the toxicity of biocide aerosols, we employed 3D reconstructed human skin epidermis, KeraSkin^TM^ [19], and a 3D airway epithelium model, SoluAirway^TM^, and exposed them to aerosolized biocides using commercially available medical nebulizers and a custom-made exposure chamber. The applied amount of biocide aerosols could be controlled by adjusting the exposure time after establishing nebulization time and deposited mass relationship. Furthermore, effects of aerosol particle sizes could be also examined by applying nebulizers producing 3 μm median mass aerodynamic diameter (MMAD) or 5 μm MMAD. With this system, we successfully evaluated dermal and airway toxicity of biocide aerosols quickly and inexpensively, which could provide a simple tool to assess the toxicity of biocide aerosols.

## 2. Materials and Methods

### 2.1. Biocidal Substances and Other Chemicals

We selected three substances, 1,2-benzisothiazol-3(2H)-one (BIT), 2-phenoxyethanol (PE), and 2-phenylphenol (OPP) that showed a concentration-dependent rate of tissue viability. Three biocides used in this experiment were purchased from Sigma-Aldrich (St. Louis, MO, USA). Experimental concentrations of biocides were determined in consideration of solubility and maximum permitted limits in Korea and abroad regulation (Table 1). MTT ((3-[4, 5-dimethylthiazol-2-yl]-2, 5-diphenyl-tetrazolium bromide) was purchased from Sigma-Aldrich (St. Louis, MO, USA) for measuring tissue viabilities.

### 2.2. 3D Reconstructed Human Epidermis Model (KeraSkin^TM^)

A reconstructed human epidermis model (KeraSkin^TM^) and KeraSkin^TM^ culture media were purchased from Biosolution Co., Ltd. (Seoul, Korea). KeraSkin^TM^ was placed on a six-well plate filled with 0.9 mL of culture medium provided by the manufacturer per a well and pre-incubated overnight at 37 °C in a humidified atmosphere conditioned with 5% CO_2_. Skin irritation test was conducted according to Organisation for Economic Co-operation and Development (OECD) test guideline 439 [20]. After pre-incubation, tissues were treated with 40 μL of biocides dissolved in 1% dimethyl sulfoxide (DMSO) in phosphate-buffered saline (PBS). After 30 min incubation, tissues were gently rinsed with warmed PBS and then further incubated for 42 h. PBS including 1% DMSO was used as a negative control, and the positive control was 5% sodium dodecyl sulfate. Control tissues followed the same schedule as biocide-treated tissues.

### 2.3. 3D Reconstructed Human Airway Mucosa Model (SoluAirway^TM^)

A reconstructed human airway mucosa model, SoluAirway^TM^, and SoluAirway^TM^ culture media were purchased from Biosolution Co., Ltd. (Seoul, Korea), which is a generic model of EpiAirway™ (MatTek, Ashland, MA, USA). Airway tissue irritation was evaluated according to a previous study with a minor modification [21]. SoluAirway^TM^ was placed on a six-well plate filled with 0.9 mL of SoluAirway^TM^ culture media per well and pre-incubated overnight at 37 °C in a humidified atmosphere conditioned with 5% CO_2_. Then, stabilized tissues were treated with 100 μL of biocides (1% DMSO in distilled water). After the aerosol application, tissues were placed on a 24-well plate and incubated for 3 h. Then, the apical surface of tissues was gently rinsed four times with 0.4 mL of warmed PBS to remove all chemicals from the surface. Control tissues were treated with distilled water including 1% DMSO (negative control) or 14.7 mg/mL formaldehyde (positive control) purchased from Sigma-Aldrich. All solution treated to tissues contained 1% DMSO.

### 2.4. Aerosol Generation and Application

**Figure 1:**
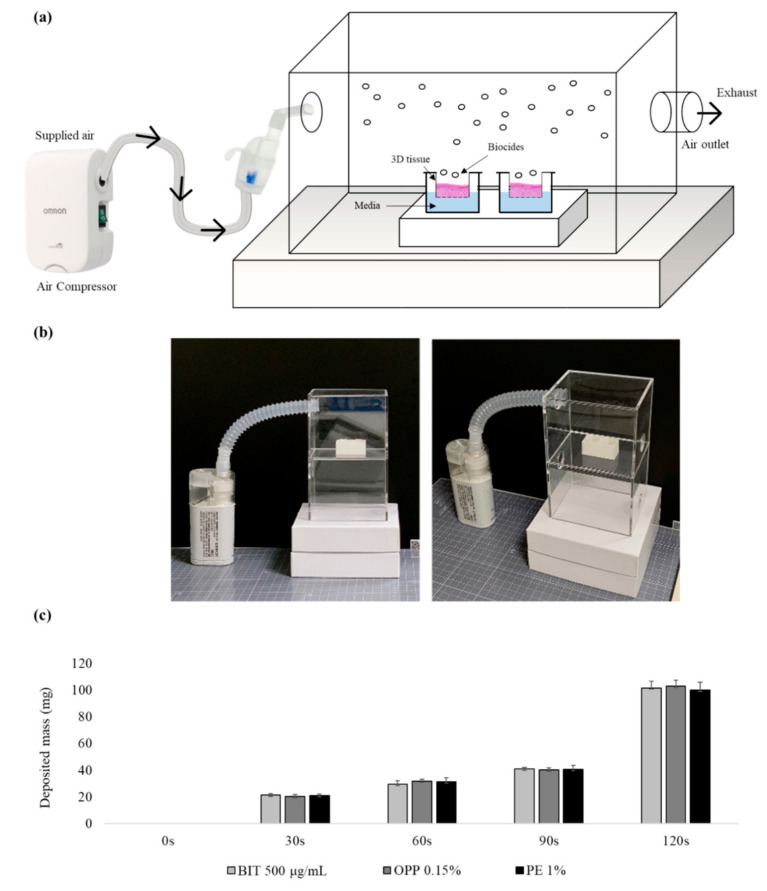
Aerosol exposure system and time course in the mass deposition of aerosolized biocides: (**a**) Schematic representation and (**b**) photo of aerosol application. Aerosol exposure occurs in the middle of the chamber. The tissue inserts are housed in a tissue tray prefilled with culture media during exposure. (**c**) The time course in the mass deposition of aerosolized biocides. Data show mean mass deposited during exposure time (mean ± SD, *n* = 4). Biocides at other concentrations also showed similar trends (data not shown).

We used two medical nebulizer models, NE-U150 (a mesh-sonication type) and NE-C803 (a jet-nebulization type, OMRON healthcare, Kyoto, Japan), to expose KeraSkin^TM^ and SoluAirway^TM^ tissues to aerosolized biocides. NE-U150 sprays aerosol with median mass aerodynamic diameter (MMAD) of 5 μm and NE-C803 generates with MMAD of 3 μm. The amount of the exposed aerosol depends on the nebulization rate and the type of materials. In order to apply the designated amount of biocides, the biocides applied on the tissue were weighed over a 30 s time interval (OHAUS microbalance, Newark, NJ, USA). In a preliminary test, we confirmed that the density of the biocides solution (in distilled water, DW) was found to be about 1.0. The applied amounts of all three biocides, BIT, PE, and OPP, were about 40 mg on average when nebulized for 90 s and 100 mg for 120 s with NE-U150 (5 μm MMAD size aerosol). With NE-C803 (3 μm MMAD aerosol), it took ~4 min to apply 100 mg of biocides. For the aerosol application, the tissues were placed inside the chamber (10 × 8 × 8 cm acryl box) on a well-tissue tray. Experimental designs of aerosol chamber and the time course of the deposited mass are presented in Figure 1. To prevent the tissue from drying, 200 μL of culture media was pre-filled in the tray. Since the aerosol exposure was completed within 4 min, this procedure was conducted in the chamber under a chemical hood.

### 2.5. Analysis of Biocides in the Aerosol

#### 2.5.1. LC-MS/MS Analysis

The sprayed aerosols were collected, and the concentration of biocides was analyzed referring to the previous studies [22,23]. BIT and OPP were analyzed by high performance liquid chromatography (Agilent 1200 series HPLC; Agilent Technologies, Santa Clara, CA, USA) coupled with triple quadrupole mass spectrometer (EVOQ Qube™; Bruker Daltonics, Billerica, MA, USA) with C18 column (ZORBAX Eclipse Plus C18; 2.1 mm × 50 mm, 1.8 μm; Agilent Technologies, Santa Clara, CA, USA). The column temperature was kept at 40 °C. The injection volume was 1 μL. 

When analyzing the amount of BIT, the mobile phase A was 0.1% formic acid in deionized water, while mobile phase B was 100% methanol, and mobile phase flow rate was 0.3 mL/min. The gradient started at 30% of solvent B for 5 min then changed linearly to 100% of solvent B in 10 min and maintained until 13 min. Then, the gradient set back to the initial percentage of solvent B (30%) after 15 min of LC run, and it was maintained for 5 min for equilibrium. A heated electrospray ionization (HESI) source was used to obtain MS spectra, and the ion source parameters were as follows: spray voltage, 4500 V; cone temperature, 350 °C; cone gas flow, 20 psi; heated probe temperature, 250 °C; probe gas flow, 45 psi; nebulizer gas flow, 55 psi. Sample introduction and ionization was electrospray ionization in the positive ion mode. Multiple reaction monitoring (MRM) mode was used for quantitative analysis of BIT, and the mass transition ion pairs were selected as m/z 152.5 → 109.1 (15.0 V) and 152.0 → 133.8 (22.0 V). 

When analyzing OPP, the mobile phase A was 50 mM ammonium acetate in deionized water, while mobile phase B was 100% methanol, and mobile phase flow rate was 0.35 mL/min. The gradient started at 10% of solvent B for 5 min then changed linearly to 100% of solvent B in 10 min and maintained until 15 min. Then, the gradient set back to the initial percentage of solvent B (10%) after 17 min of LC run, and it was maintained for 6 min for equilibrium. Sample introduction and ionization was electrospray ionization in the negative ion mode, and the spray voltage was –4000 V. The mass transition ion pairs were selected as m/z 169.0 → 115.1 (27.0 V) and 169.0 → 141.1 (26.6 V). All other HPLC and MS/MS conditions were set the same as for BIT analysis. 

#### 2.5.2. GC-MS Analysis

PE was analyzed by gas chromatography (7890B; Agilent Technologies, Santa Clara, CA, USA) coupled with quadrupole mass spectrometer (5977A; Agilent Technologies, Santa Clara, CA, USA) with capillary column (HP-5ms UI; 30 m × 0.25 mm, 0.25 μm; Agilent Technologies, Santa Clara, CA, USA). Samples were injected 1 µL in splitless mode, and injector temperature was 280 °C. The carrier gas was high purity helium with a flow of 1.0 mL/min. The initial column temperature was 60 °C and held 3 min and then ramped to 300 °C at the rate of 30 °C/min and held 10 min. The ionization was carried out in the electron impact (EI) mode at 70 eV. The transfer line and the ion source temperatures were maintained at 280 °C and 250 °C, respectively. Data were obtained in the selected ion monitoring (SIM). The quantifier ion and the qualifier ion were m/z 94 and m/z 138, respectively. 

#### 2.5.3. Analytical Validation

Analytical methods developed above were validated for linearity, recovery, reproducibility, limit of detection (LOD), and limit of quantitation (LOQ). Standard calibration solutions for BIT, OPP, and PE prepared from 15.625 to 250 ng/mL were used for the establishment of calibration curves. R^2^ value was more than 0.9995 for all the analytes, confirming the linearity. Recovery was from 80 to 110% for three concentrations, 10, 20, and 40 ng/g samples with SD within 10%, supporting the reproducibility. LOD and LOQ for BIT and OPP were 7.81 ng/mL and 15.625 ng/mL, respectively. For PE, LOD and LOQ were 1.95 ng/mL and 3.91 ng/mL. 

### 2.6. MTT Assay

Tissue viability was measured by the cellular reduction of MTT (3-(4,5-dimethlthiazol-2-yl)-2,5-diphenyltetrazolium bromide). MTT was reduced to a dark blue insoluble formazan by mitochondrial reductase, which could be extracted and measured with optical density at 570 nm. After post-incubation (for KeraSkin^TM^) or treatment (for SoluAirway^TM^), tissues were transferred to 24-well culture plates containing 200 μL of MTT (KeraSkin^TM^; 0.4 mg/mL, SoluAirway^TM^; 1.0 mg/mL) diluted in sterile PBS and incubated for 3 h at 37 °C and 5% CO_2_. Following incubation, KeraSkin^TM^ tissues were transferred to a new 6-well plate prefilled with 2 mL of isopropanol. Formazan extraction was performed at room temperature for 3 h, protected from the light. For SoluAirway^TM^, tissues were submerged in 2.0 mL isopropanol, in which all tissues were maintained overnight at room temperature. Following procedures, 200 μL extractant from each tissue was transferred to a 96-well plate, and optical density (OD) was measured at 570 nm using isopropanol as a blank with a microplate spectrophotometer at 570 nm (BioTek Instruments, Inc., Winooski, VT, USA) Viability for each tissue was calculated with the optical density (OD) relative to negative control according to the following equation: relative viability = [OD _test tissue_ ÷ mean OD _negative control_] × 100.

### 2.7. Histological Analysis

For the histological analysis, all samples were trimmed 10 mm width off the hem of the tissues and fixed in 4% phosphate-buffered formalin (PFA) for 24 h. Fixed samples were sealed with paraffin films and cut into 5 μm sections using microtome (Leica, RM2235), followed by hematoxylin-eosin staining. The sections were stained with Gill 3 hematoxylin (HX87960674) for 7 min 30 s and 0.5% eosin in 95% EtOH. After staining with hematoxylin and eosin, the stained tissues were washed immediately and sequentially proceeded as follows: dip in distilled H_2_O until eosin stops streaking, 50% EtOH and 70% EtOH for 10 times, sequentially. Then, they were incubated in 95% EtOH for 30 s and 100% EtOH for 1 min. The incubated samples were covered with a mounting solution (Thermo Scientific, 6769007) and examined under the light microscope (OLYMPUS, BX43).

### 2.8. Statistical Analysis

Data are expressed as the mean ± SD. Difference from vehicle control was analyzed using Student *t*-test. *p*-values of 0.05 or less were considered significant.

## 3. Results

### 3.1. The Local Toxicity of Biocides after Direct or Aerosol Exposure on the Reconstructed Human Skin Epidermis Model, KeraSkin^TM^

**Figure 2:**
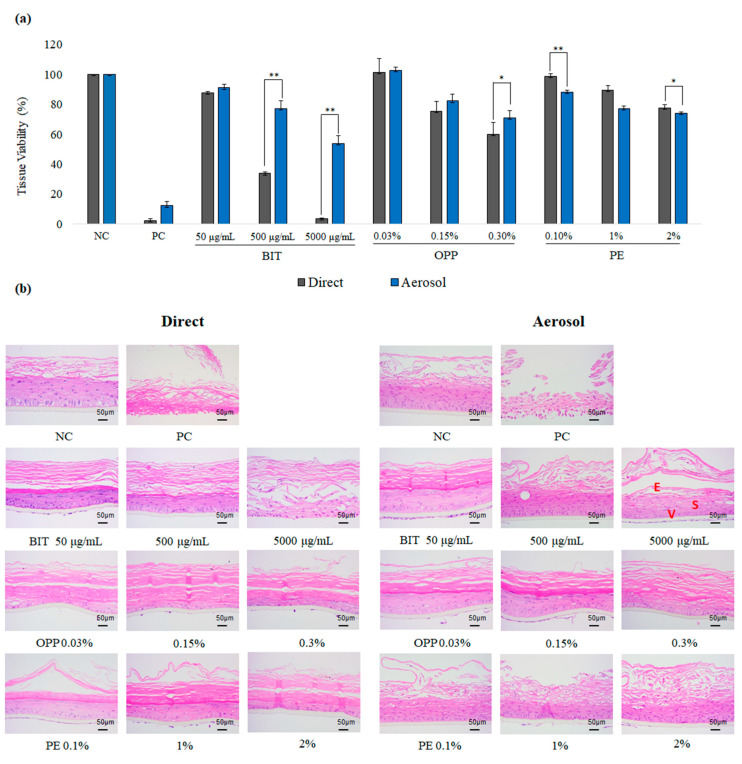
Toxicity of biocides on KeraSkin^TM^ after direct or aerosol application. (**a**) Tissue viability of KeraSkin^TM^ tissue measured with ((3-[4, 5-dimethylthiazol-2-yl]-2, 5-diphenyl-tetrazolium bromide) (MTT) assay at 48 h after the exposure to biocides through direct (grey bars) or aerosol application (blue bars) for 30 min. Values are mean ± SD (*n* = 3). NC; negative control (1% DMSO in PBS), PC; positive control (5% sodium dodecyl sulfate); BIT; 1,2-Benzothiazol-3-one, PE; 2-Phenoxyethanol, OPP; 2-Phenylphenol. * *p* < 0.05 or ** *p* < 0.01 by Student *t*-test. (**b**) Representative histological photographs of the treated tissues after hematoxylin-eosin (H&E) staining. Scale bar is 50 µm. E, V, and S stand for erosion (detachment of epithelial cells), vacuolation (formation of vacuoles in cytosol), and spongiosis (intercellular edema), which was indicated on BIT 5000 μg/mL photo.

To evaluate the local toxicity of aerosolized biocides on the skin, the reconstructed human skin epidermis model KeraSkin^TM^ was employed. Biocides were aerosolized with a medical nebulizer (model NE-U150) to 5 μm MMAD aerosol and applied on the KeraSkin^TM^ tissue such that the final applied amount would be 40 mg (40 μL in volume. see Material and Methods for more details). Then, the tissues were incubated for 30 min and washed thoroughly. The resulting tissues were further incubated for 48 h, and the tissue viability was measured by MTT assay (Figure 2a). For comparison, 40 mg of biocides were directly applied on the KeraSkin^TM^ and followed the same procedure. As a result, the tissue viabilities treated with aerosolized BIT and OPP were generally higher than direct application. The difference in the local toxicity of biocides was pronounced for BIT. In contrast, there was only small difference in the viabilities after aerosol from direct application for PE or PC (5% SDS). The histological examination of the treated tissues also confirmed the MTT viability data (Figure 2b). The tissues directly exposed to BIT and OPP showed more severe damages than those to aerosols such as erosion, vacuolation, spongiosis, and necrosis, suggesting that BIT and OPP may be less toxic to the skin when applied in aerosol.

### 3.2. Comparison of Local Toxicity of Biocides after Direct or Aerosol Exposure on the Reconstructed Human Upper Airway Model, SoluAirway^TM^

**Figure 3:**
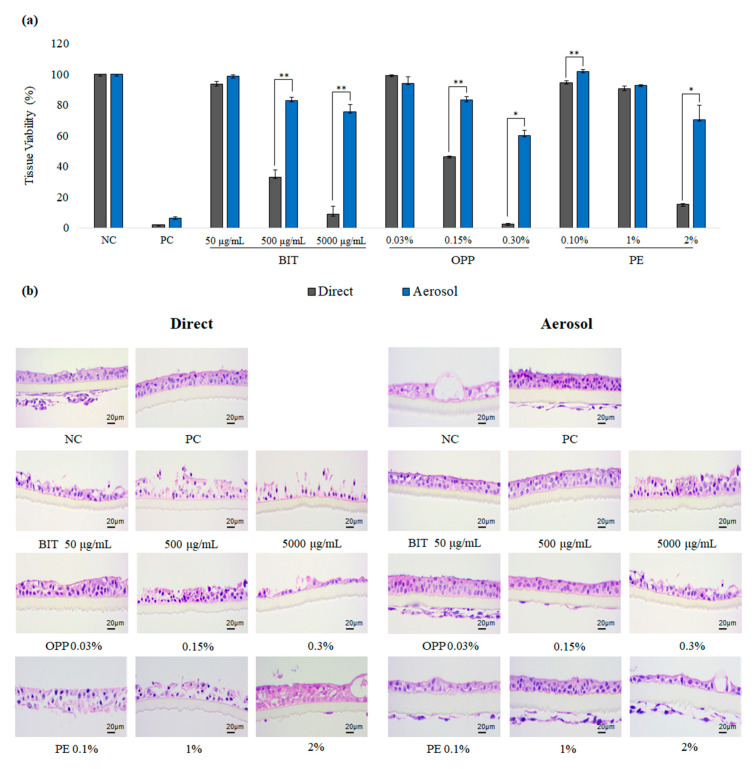
Toxicity of biocides on SoluAirway^TM^ after direct or aerosol application: (**a**) Tissue viability of SoluAirway^TM^ tissue measured with MTT assay after the exposure to biocides through direct (grey bars) or aerosol application (blue bars) for 3 h. Values are mean ± SD (*n* > 2) or ½difference (*n* = 2). NC; negative control (phosphate-buffered saline, PBS), PC; positive control (14.7 mg/mL formaldehyde) by Student *t*-test. (**b**) Representative histological photographs of the treated tissues after H&E staining. Scale bar is 20 µm. Cell debris below the transmembrane shall be ignored since they were generated during tissue section.

To evaluate the local toxicity of aerosolized biocides on the airway, the reconstructed human upper airway model SoluAirway^TM^ was employed. As was similar with the skin epidermis experiment, BIT and OPP were less toxic to SoluAirway^TM^ when applied in aerosol (Figure 3a). Of note, this pattern was pronounced, and even PE aerosol, which showed similar degree of toxicity with direct application in the skin epidermis, showed reduced toxicity in SoluAirway^TM^. In the histological examination, it could be confirmed that aerosolized biocides resulted in less severe damages on SoluAirway^TM^ than directly applied biocides (Figure 3b).

### 3.3. Concentrations of Biocides after Aerosolization

We speculated that, during the aerosolization, biocides may be degraded, resulting in decreased concentrations of biocides in the aerosol, which may account for the reduced toxicity of aerosolized biocides (Table 2). The aerosolized biocides were collected and analyzed for the concentrations of biocides. As a result, it was confirmed that the concentrations of all three biocides decreased after the aerosolization at all concentrations. These patterns became more pronounced as the concentrations of biocides were higher, indicating that significant amounts of biocides were lost during aerosolization.

### 3.4. Effects of Aerosol Particle Sizes on the Local Toxicity of Biocides on SoluAirway^TM^

**Figure 4:**
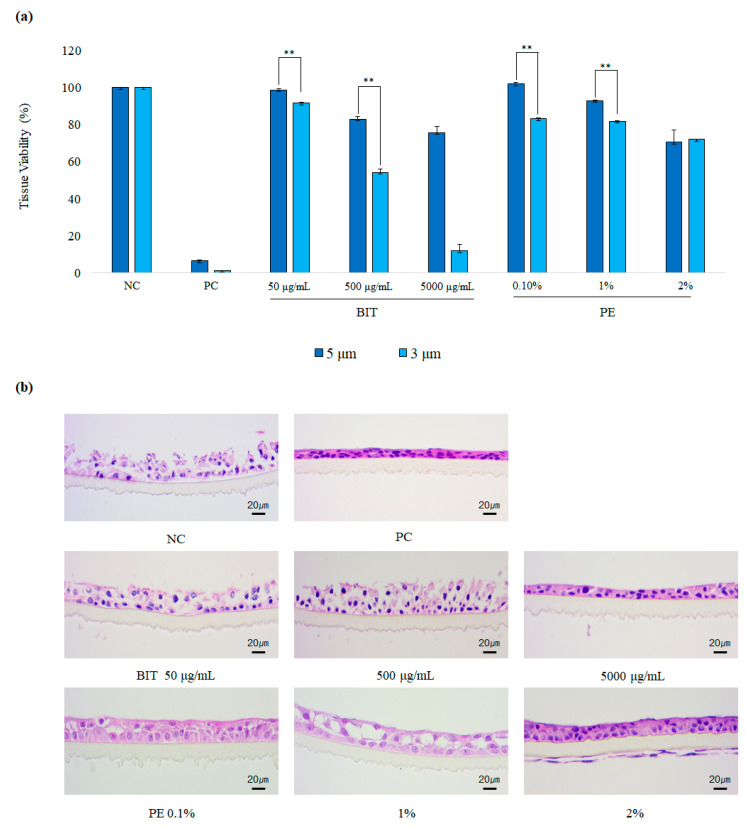
Toxicity of BIT and PE on SoluAirway^TM^ after the application of 3 µm or 5 μm aerosol: (**a**) Tissue viability of SoluAirway^TM^ tissue measured with MTT assay after the exposure to aerosols of BIT and PE with 5 µm MMAD (blue) or 3 µm MMAD (light blue) for 3 h. Values are mean ± SD (*n* > 2) or ½difference (*n* = 2). ** *p* < 0.01 by Student *t*-test (**b**) Representative histological photographs of the treated tissues after H&E staining. Scale bar is 20 µm.

Particle size is considered as a key determinant of aerosol toxicity. Decrease in particle size promotes the aerosol dispersion during the first seconds of inhalation. Additionally, the smaller aerosol particles can penetrate deeper into smaller airways of human respiratory tract and can subsequently be deposited more efficiently than bigger ones [24]. However, it is not known whether the aerosol size itself can affect the local toxicity of biocides. Using SoluAirway^TM^, we examined the effects of aerosol particle size on the local toxicity of biocides. Soluairway^TM^ was exposed to aerosols of BIT and PE, which showed the largest and the smallest differences in toxicity between direct and aerosol applications, respectively, at two different MMADs, 3 μm or 5 μm, and the tissue viability was determined. Of note, the local toxicity of aerosolized biocides BIT and PE with MMAD 3 μm was stronger than that of 5 μm (Figure 4). These patterns became more evident at higher concentrations, indicating that the aerosol size itself may affect the toxicity of biocides as well as the dispersion characteristics.

## 4. Discussion

Here, we examined the impact of aerosolization on the local toxicity of biocides on the reconstructed human skin epidermis and the airway tissue models. Reconstructed human tissue models provide more physiologically relevant conditions than conventional 2D cell experiments [25]. Using 3D reconstructed tissue models, we evaluated the local toxicity of three biocides, BIT, PE, and OPP, widely used in commercial products on the skin and the airway. Compared to direct exposure, aerosol exposure of biocides resulted in weaker toxicity on skin epidermis and airway tissue models in general even though the same mass dose of biocides was applied on the tissues. We demonstrated that, during aerosolization, the concentrations of biocides in aerosols may decrease, which accounts for the weaker toxicity of aerosolized biocides. Of note, we found that aerosols with smaller particle sizes (MMAD) were more toxic to the airway tissue model than those with greater MMADs, demonstrating that aerosol particle size may be an important factor for the toxicity of biocides.

Here, we found that the direct exposure of biocides inflicted stronger toxicity on skin and airway, providing an important implication for animal intra-tracheal instillation tests. Animal intra-tracheal instillation study has been widely used to investigate the respiratory toxicity of chemicals instead of the standard animal inhalation test [26]. In contrast to the standard animal inhalation test, which needs nose-only or whole body exposure systems, intra-tracheal instillation needs only a device to accurately deliver the designated dosage of test chemicals directly to the lung, saving a lot of cost and time to examine the inhalational toxicity of test chemicals [27]. However, intra-tracheal intubation often over-predicts the toxicity of test chemicals compared to the standard animal inhalation test [28]. The discrepancy between inhalation test and intra-tracheal test was explained by the acuteness of the dosing and the deeper deposition of the test chemicals in intra-tracheal dosing [29]. Adding to this explanation, we could show that aerosolization of test chemicals may lower the active ingredient concentration, resulting in reduced toxicity. To correctly evaluate the toxicity of aerosolized biocides, the disposition of biocides during aerosolization must be taken into consideration. In this context, the intra-tracheal instillation may not be appropriate for the study of test chemicals that can become lost during the aerosolization [30], or, at least, the disposition of biocides during aerosolization must be analyzed and taken into consideration for the dosage adjustment.

Recently, an airway tissue model was employed to assess the risk of aerosolized pesticides [31]. In this study, the dose–toxicity curve was established using an airway tissue model after the direct application of the pesticide. The computational fluid dynamics (CFD) model was used to estimate the deposition of aerosolized pesticide to the respiratory tract for the calculation of the safety margin [32,33]. According to our results, direct exposure may over-estimate the toxicity of biocides. Establishment of a dose–toxicity curve with the aerosolized pesticide would provide more accurate estimation of the risk of aerosolized chemicals. Namely, application of the deposited mass of the biocides’ aerosol estimated with the CFD model on the airway model would present a more realistic exposure scenario. 

We demonstrated that aerosol particle size may affect the toxicity of biocides. It is well-known that aerosols with smaller particle sizes are more toxic to the lung [34]. In a study using cadmium chloride (CdCl_2_) as a model for toxic aerosol particles, rats exposed to 33 nm particles showed the highest level of respiratory toxicity, followed by animals exposed to 637 nm particles and by those exposed to 1495 nm particles [35]. The higher toxicity of aerosols with smaller particle sizes has been explained by the greater deposition in the lower respiratory tract and larger retention in the lung due to the aerodynamic of particulate matters [36]. Furthermore, we demonstrated that, even though the deposited mass amount of aerosolized biocides on the airway tissues was the same, the toxicity of aerosols with smaller sizes was significantly stronger, suggesting that there may be factors other than macroscopic aerodynamics. The reason behind the differences in toxicity between 3 μm aerosol and 5 μm aerosol may stem from the differences in the concentrations of biocides in the aerosols or the contact of biocide aerosols on the tissues. We found that BIT aerosols with 3 μm size were with higher BIT concentrations than those with 5 μm size, but the contents of PE in 3 μm aerosols were lower than 5 μm, suggesting that other factors such as tissue contact may have contributed, although further studies are necessary to confirm it. Additionally, it would be important to identify the disposition and the behavior of the biocides during aerosolization, which shall be addressed by more sophisticated assays such as a radiotracer study.

Here, we demonstrated that, with respect to potency of toxicity, BIT was the strongest, followed by OPP and PE. BIT is used as a preservative with a maximum concentration of 0.05% in the EU, the US, and Canada [37]. OPP (0.15%) and PE (1%) are allowed to be used as preservatives in limited concentrations in cosmetics in South Korea and other countries [38]. Since there is only limited information on the animal toxicity of these biocides, it is difficult to compare the toxicity of these biocides. However, the order of toxic potency of BIT, OPP, and PE observed in our study exactly matches the order of their permitted concentrations, supporting that our experimental models may reflect the biocide toxicity relatively well, although further studies are necessary.

In summary, using human epidermis and airway tissue models along with a simple aerosol exposure system, we demonstrated that aerosol exposure of biocides may induce weaker toxicity on the skin or the airway compared to direct exposure, which appears to be attributable to decreased concentrations of biocides in aerosols. We also found that the biocide aerosols with smaller particle sizes were per se more toxic to the airway tissue model than those with greater particle sizes. We believe that our in vitro test system may be useful for the toxicity evaluation of various forms of aerosolized test chemicals with respect to time, cost, and animal welfare.

## Figures and Tables

**Table 1:** Maximum permitted concentration of three biocides tested.

Chemical	International Union of Pure and Applied Chemistry (IUPAC) Name	Maximum Permitted Concentration
BIT	1,2-Benzothiazol-3-one	0.05% in EU and USA
PE	2-Phenoxyethanol	Cosmetics, 1%
OPP	2-Phenylphenol	Cosmetics, 0.15% (as phenol)

BIT: 1,2-benzisothiazol-3(2H)-one; PE: 2-phenoxyethanol; OPP: 2-phenylphenol.

**Table 2:** Analysis of biocides in the deposited aerosols (5 μm and 3 μm median mass aerodynamic diameter (MMADs)) with LC/MSMS and GC/MS.

Biocides	Indicated Conc.	After Nebulization	
		5 μm	3 μm
BIT (1,2-Benzisothiazol- 3(2H)-one)	50 μg/mL	37.34 ± 19.85	44.70 ± 6.00
	500 μg/mL	394.39 ± 47.71	457.8 ± 86.73
	5000 μg/mL	1144.11 ± 134.31	857.0 ± 69.18
OPP (2-phenylphenol)	0.03%	0.006 ± 0.004	0.021 ± 0.017
	0.15%	0.027 ± 0.01	0.103 ± 0.013
	0.3%	0.048 ± 0.019	0.099 ± 0.009
PE (2-phenoxyethanol)	0.1%	0.04 ± 0.002	0.028 ± 0.012
	1%	0.49 ± 0.059	0.251 ± 0.099
	2%	1.21 ± 0.259	0.523 ± 0.172

## Data Availability

The data presented in this study are available on request from the corresponding author. The data are not publicly available since they are raw data.
